# X‐ray computed tomography and its potential in ecological research: A review of studies and optimization of specimen preparation

**DOI:** 10.1002/ece3.4149

**Published:** 2018-07-06

**Authors:** Yeisson Gutiérrez, David Ott, Mareike Töpperwien, Tim Salditt, Christoph Scherber

**Affiliations:** ^1^ Institute of Landscape Ecology University of Münster Münster Germany; ^2^ Institute for X‐Ray Physics University of Göttingen Göttingen Germany

**Keywords:** μ‐CT, anatomy, animal imaging, computed tomography, internal morphology

## Abstract

Imaging techniques are a cornerstone of contemporary biology. Over the last decades, advances in microscale imaging techniques have allowed fascinating new insights into cell and tissue morphology and internal anatomy of organisms across kingdoms. However, most studies so far provided snapshots of given reference taxa, describing organs and tissues under “idealized” conditions. Surprisingly, there is an almost complete lack of studies investigating how an organism′s internal morphology changes in response to environmental drivers. Consequently, ecology as a scientific discipline has so far almost neglected the possibilities arising from modern microscale imaging techniques. Here, we provide an overview of recent developments of X‐ray computed tomography as an affordable, simple method of high spatial resolution, allowing insights into three‐dimensional anatomy both *in vivo* and *ex vivo*. We review ecological studies using this technique to investigate the three‐dimensional internal structure of organisms. In addition, we provide practical comparisons between different preparation techniques for maximum contrast and tissue differentiation. In particular, we consider the novel modality of phase contrast by self‐interference of the X‐ray wave behind an object (i.e., phase contrast by free space propagation). Using the cricket *Acheta domesticus* (L.) as model organism, we found that the combination of FAE fixative and iodine staining provided the best results across different tissues. The drying technique also affected contrast and prevented artifacts in specific cases. Overall, we found that for the interests of ecological studies, X‐ray computed tomography is useful when the tissue or structure of interest has sufficient contrast that allows for an automatic or semiautomatic segmentation. In particular, we show that reconstruction schemes which exploit phase contrast can yield enhanced image quality. Combined with suitable specimen preparation and automated analysis, X‐ray CT can therefore become a promising quantitative 3D imaging technique to study organisms′ responses to environmental drivers, in both ecology and evolution.

## 1 INTRODUCTION

Organisms respond to environmental drivers in a variety of ways, including changes in behavior, morphology, growth, or reproduction. Advances in imaging technology across scales have opened up new opportunities to estimate reproduction or to measure changes in morphology. Changes in internal morphology (such as complexity of neural tissues) are among the fastest (and often plastic) responses to environmental drivers, often preceding future changes in behavior or reproduction. Being able to fast‐track or even predict such responses will allow novel insights into physiological, behavioral, and evolutionary ecology.

A variety of automated techniques (such as confocal laser scanning microscopy, light sheet microscopy, nuclear magnetic resonance imaging, and microcomputed tomography) are available to study internal morphology or organisms, but these frequently require manual processing of low‐contrast regions in every section of the complete tissue, which precludes processing large numbers of samples. In contrast, classic histology (microtomy and episcopic microscopy) allows a variety of stains for tissue recognition, but requires destruction of the samples.

Yet, recent years have seen the development of novel imaging techniques such as microcomputed tomography (μ‐CT) that overcome these problems, allowing unprecedented insights into cell and tissue morphology and internal anatomy of organisms across kingdoms, from bacteria to vertebrates (Dhondt, Vanhaeren, Van Loo, Cnudde, & Inzé, 2010; Stender et al., 2014; Wipfler, Pohl, Yavorskaya, & Beutel, 2016). Fields such as taxonomy (Akkari, Enghoff, & Metscher, 2015; Faulwetter, Vasileiadou, Kouratoras, Dailianis, & Arvanitidis, 2013; Fernández, Kvist, Lenihan, Giribet, & Ziegler, 2014) and morphology (Mattei, Riccio, Avila, & Wolfner, 2015; Wipfler et al., 2016) have greatly benefited from μ‐CT technique. However, most studies to date have focused on in‐depth studies of single individuals, and organisms′ responses to environmental drivers were only rarely considered.

The effects of external drivers (such as global change components) on organisms can be studied by investigating the response of individuals (behavior, morphology, physiology) or populations (reproduction, survivorship) (Bale et al., 2002; Bidart‐Bouzat & Imeh‐Nathaniel, 2008). Morphological changes in response to environmental drivers are usually studied within the framework of trait‐based ecology (Deraison, Badenhausser, Loeuille, Scherber, & Gross, 2015). However, the traits considered are often related to external morphology or behavior, rather than to internal morphology of organisms.

Recently, studies (e.g., in pollinator ecology) have started to use modern imaging techniques, for example, to assess changes in brain morphology of bees and butterflies in response to environmental and social stimuli (Jones, Leonard, & Papaj, 2013; Maleszka, Barron, Helliwell, & Maleszka, 2009; Snell‐Rood, Papaj, & Gronenberg, 2009).

Here, we provide an overview of ecological studies using X‐ray CT, to study the three‐dimensional external and internal structures of organisms. In addition, we experimentally study a range of staining and fixation approaches useful for future studies and provide an outlook into questions that might be answered using micro‐CT in the future. Finally, we propose to especially exploit phase contrast, which has now become a reality also with advanced laboratory μ‐CT (Bartels, Hernandez, Krenkel, Moser, & Salditt, 2013; Töpperwien, Krenkel, Quade, & Salditt, 2016; Töpperwien et al., 2017)

## 2 TECHNIQUES FOR TOMOGRAPHIC RECONSTRUCTIONS

**Figure 1:**
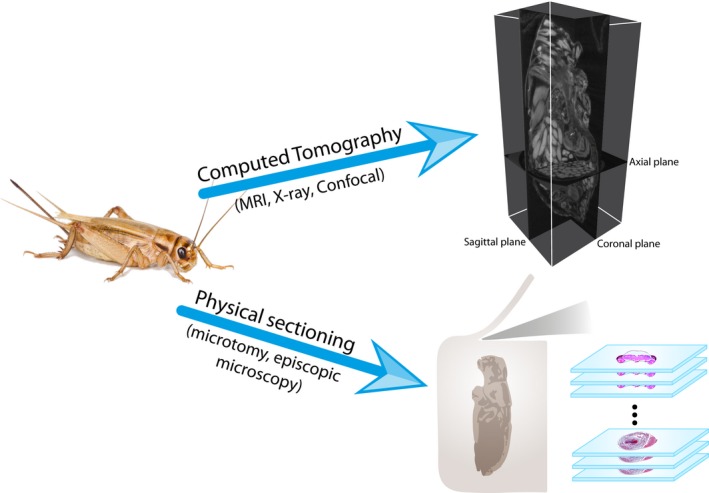
Conceptual figure showing procedural differences between computed tomography and physical sectioning of samples. CT (by means of several techniques) does not require sample destruction, and the resolution is identical in all orientations (isotropy) and enables visualization in different angles or axes. On the other hand, physical sectioning allows a wider variety of dying techniques for tissue recognition, but the plan is determined by the orientation of the sample; thus, also the z‐axis possesses a different (usually lower) resolution

Tomography refers to imaging by sections or slices through a solid object, which can be achieved through several methodologies—usually requiring different sample preparations. This approach stands out because it allows imaging entire specimens (Jasanoff & Sun, 2002), does not require sample destruction (i.e., it is noninvasive), avoids tissue deformation (i.e., it retains natural stereogeometry), and is time‐saving (Jährling, Becker, Schönbauer, Schnorrer, & Dodt, 2010; Smith et al., 2016; Sombke, Lipke, Michalik, Uhl, & Harzsch, 2015). Tomographic imaging can be particularly advantageous in studies that require several assessments of the same individuals over time, as in developmental biology (Goodman & Chudekt, 1995; Hart, Bowtell, Köckenberger, Wenseleers, & Ratnieks, 2003), or when sectioning the same sample along different angles or axes is needed (Figure 1).

One of the most interesting aspects of tomography is the possibility to generate three‐dimensional (3D) models. Such models, for example, have become popular within the area of plant phenotyping, where crop plant varieties are now routinely screened using a broad spectrum of imaging approaches (Fiorani & Schurr, 2013). Using classical histological procedures (e.g., microtomy and episcopic microscopy), such reconstructions can only be achieved going through every layer and manually selecting and aligning the desired tissue or organ, thereby commonly over‐ or underestimating the volume of soft tissues due to the lack of isotropic resolution (e.g., poor resolution in the *z*‐axis) (Sickert, Rodner, & Denzler, 2015; Smith et al., 2016). In contrast, with 3D imaging techniques, in particular μ‐CT, automatic surface (Friedrich & Beutel, 2008) and volume reconstructions can be quickly carried out, allowing to accurately determine surface areas and volumes for comparative studies (Hart et al., 2003), in addition to be visually attractive and self‐explanatory.

Apart from X‐ray CT, the most commonly used techniques for tomographic reconstructions are nuclear magnetic resonance imaging (MRI). Other techniques, such as ultramicroscopy, confocal laser scanning microscopy (CLSM), or light sheet microscopy, also allow high resolution down to the submicrometer range without physically sectioning the samples (Becker, Jährling, Kramer, Schnorrer, & Dodt, 2008). However, the latter two techniques are based on transmission of visible light (Jährling et al., 2010) and require the sample to go through a chemical clearance process. Moreover, these techniques are limited to tissues thinner than 500 μm, requiring elaborated sample preparation (Sombke et al., 2015).

MRI and X‐ray CT do not require complex sample manipulation, and can even be performed *in vivo* (Callaghan, 1991; Hart et al., 2003; Jasanoff & Sun, 2002), although this could compromise the quality of the images because of internal movements of organs and fluids (Hart et al., 2003). While some studies suggest that low doses (<500 Gray) of radiation have only negligible effect on insects survivorship, the long‐term effects of X‐rays on insects have remained poorly studied so far (Socha, Westneat, Harrison, Waters, & Lee, 2007; Westneat, Socha, & Lee, 2008).

Primarily due to the much higher spatial resolution of X‐ray CT, we consider it as a particularly well‐suited choice for future studies. Samples are certainly more easily prepared for MRI than X‐ray CT, in particular for those studies which require metal‐based staining for soft tissue imaging (Metscher, 2009a, 2013). Nevertheless, magnetic resonance scanning systems are limited in resolution (Metscher, 2013; Metzner et al., 2015) and often have prohibitive costs of operation (Ziegler et al., 2011). Furthermore, air spaces routinely found in biological samples can cause artifacts in MRI (Jasanoff & Sun, 2002; Wecker, Hörnschemeyer, & Hoehn, 2002; Ziegler et al., 2011).

## 3 X‐RAY‐BASED COMPUTED TOMOGRAPHY (X‐RAY CT AND μ‐CT)

The difference between X‐ray CT and μ‐CT is merely the level of detail: μ‐CT works at the micrometer range (Medical Subject Headings—MeSH) and has become an invaluable tool in the study of several organs and organ systems in arthropods (see a review in Westneat et al., 2008; Metscher, 2013; Sombke et al., 2015) and other invertebrates (Carbayo & Lenihan, 2016; Fernández et al., 2014). This technique allows spatial resolution in the 1–10 μm range (i.e., spanning the range from whole cells down to the level of single organelles) and a temporal resolution of less than 100 ms. Furthermore, due to recent improvements, it provides enough detail to successfully distinguish either cuticular structures or soft tissues as muscles and nervous system (Sena et al., 2015; Smith et al., 2016; Westneat et al., 2008).

Images obtained from X‐ray CT possess a homogeneous illumination with isotropic resolution at each slice, which allows consistent and precise volumetric estimates and some degree of automation of the process (Sickert et al., 2015; Smith et al., 2016). As reported by Seo, Lim, Seo, and Lee (2015), X‐ray CT is sensitive enough for internal modifications that in some cases cannot be traced through resin‐sectioned images.

More advanced variants of this technique have been demonstrated using synchrotron radiation (SR‐μCT), exploiting the high brilliance of the radiation. In practice, this can be used to generate better collimated (parallel) beams, higher flux density, and smaller bandpass by monochromatization (e.g., double silicon crystals). In combination with cone‐beam geometries, or focusing optics, submicron resolution has become possible (Hoshino, Uesugi, & Yagi, 2012; Sena et al., 2015; Westneat et al., 2008). Apart from resolution, the range of possible contrast mechanisms and levels has been significantly enhanced by use of SR. Note that by virtue of phase contrast, also nonabsorbing or weakly absorbing tissues can be visualized, based on the intrinsic phase shift which X‐rays undergo when traversing matter. In particular, phase contrast by free propagation has been exploited and has been demonstrated at submicron resolution (Cloetens et al., 1999; Lagomarsino et al., 1997; Paganin & Nugent, 1998).

Using highly focused radiation and a cone‐beam illumination geometry, a resolution range down to of 20–50 nm has even been demonstrated (Bartels, Krenkel, Haber, Wilke, & Salditt, 2015), allowing to examine details of cell organelles. Importantly, phase contrast based on free propagation is also compatible with the low partial coherence of laboratory sources, so that, subsequently, a translation from SR‐based phase‐contrast CT (SR‐PhC‐μCT) to advanced μ‐CT instrumentation was possible. The instrumental prerequisites and different geometries and phase retrieval approaches have been discussed and compared elsewhere (Bartels et al., 2013; Krenkel et al., 2015; Töpperwien, Krenkel, Müller, & Salditt, 2016; Töpperwien et al., 2017), in particular the adaptation of phase retrieval for the nonideal conditions of laboratory μ‐CT (Bartels et al., 2013; Krenkel et al., 2015; Töpperwien, Krenkel, et al., 2016; Töpperwien et al., 2017).

For this research, we have focused on studies using the more widely accessible μ‐CT versions based on laboratory radiation, both the common absorption‐based variant and the emerging phase‐contrast modality, which has also been exploited in the present experimental work.

In the context of the biological sciences, previously published reviews about X‐ray CT have focused on technical details and currently available techniques (Withers, 2007), current manufacturers and models (Schambach, Bag, Schilling, Groden, & Brockmann, 2010; for an updated list see Appendix S3), potentials and limitations of computed tomographic techniques on classical anatomy studies (Friedrich & Beutel, 2008), animal physiology (Westneat et al., 2008), and arthropod neuroanatomy (Sombke et al., 2015). To the extent of our knowledge, this is the first study addressing ecological studies involving CT for acquiring *in vivo* and *ex vivo* data.

## 4 APPLICATION OF X‐RAY COMPUTED TOMOGRAPHY IN ECOLOGICAL STUDIES

Although X‐ray CT scanners have been available since 1967 in clinics and laboratories (Hsieh, 2009), and μ‐CT has been available many years ago (Elliott & Dover, 1982; for an overview of the manufacturers see Schambach et al., 2010), few applications of these techniques in ecological studies can be found in the current literature. To date, these techniques have been more commonly used for taxonomy, phylogeny, and physiology (Beutel, Friedrich, Ge, & Yang, 2014; Fernández et al., 2014; Friedrich & Beutel, 2008; Metscher, 2013).

As a comprehensive historical analysis of the studies published on this topic was lacking, we conducted a literature search from 1974 (the date of the first paper) to 2017 using Thomson‐Reuters's ISI Web of Science (all databases). We used [“compute* tomogr*” OR “micro tomogr*” OR “micro CT”] as the primary search terms. To filter out the studies dealing with human and animal medicine and livestock production, we used secondary search terms including all animal phyla and plant and fungal divisions [Acanthocephala OR Acoelomorpha OR Annelida OR Arthropoda OR Brachiopoda OR Bryozoa OR Chaetognatha OR Chordata OR Cnidaria OR Ctenophora OR Cycliophora OR Echinodermata OR Entoprocta OR Gastrotricha OR Gnathostomulida OR Hemichordata OR Kinorhyncha OR Loricifera OR Micrognathozoa OR Mollusca OR Nematoda OR Nematomorpha OR Nemertea OR Onychophora OR Orthonectida OR Phoronida OR Placozoa OR Platyhelminthes OR Porifera OR Priapulida OR Rhombozoa OR Rotifera OR Sipuncula OR Tardigrada OR Xenacoelomorpha OR Anthocerotophyta OR Bryophyta OR Marchantiophyta OR Hepatophyta OR Lycopodiophyta OR Lycophyta OR Pteridophyta OR Pinophyta OR Coniferophyta OR Cycadophyta OR Ginkgophyta OR Gnetophyta OR Magnoliophyta OR Chytridiomycota OR Blastocladiomycota OR Zygomycota OR Glomeromycota OR Ascomycota OR Basidiomycota OR Microsporidia OR Neocallimastigomycota] and subsequently refined the search to the following research areas (in order of record count): Zoology, Environmental Sciences Ecology, Evolutionary Biology, Paleontology, Mycology, Plant Sciences, Marine Freshwater Biology, and Biodiversity Conservation.

**Figure 2:**
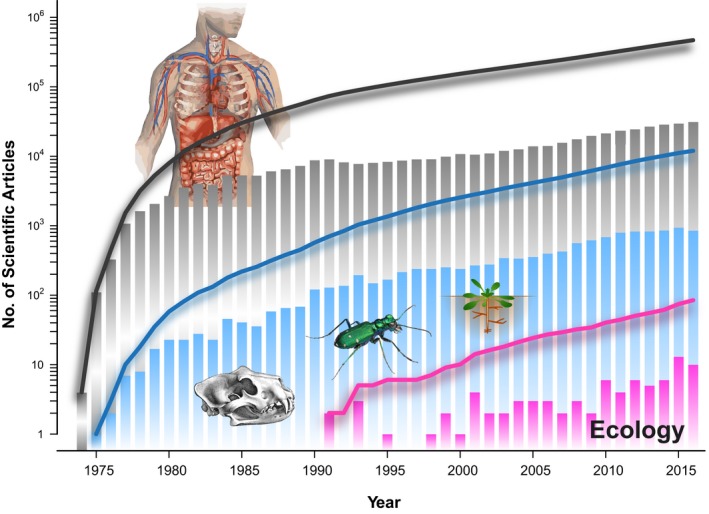
Historical analysis of the usage of x‐ray computed tomography in scientific studies by means of Thomson‐Reuters's ISI on the Web of Science (all databases). Dark gray: total number (primary search terms), blue: biological sciences (primary search terms plus secondary filtering to exclude studies dealing with human and animal medicine and livestock production), dark pink: manually refined selection of ecological studies. Bars represent the annual count of publications and lines the cumulative sum. Results showed on the *y*‐axis (presented in logarithmic scale) were obtained using a combination of search term and research areas explained in the text. Beetle image © Alex Wild, used by permission

Using only the primary search terms, we obtained 476,232 results on scientific publications, and secondary filtering resulted in 11,990 (2.52%) publications where X‐ray‐based tomography was used in the stricter sense of the natural science field biology (Figure 2). Because computed scanners were developed for medical applications, most of the obtained studies were published on related research areas—and single papers have reached up to 6,000 citations. Other outstanding fields represented in our results are engineering, mathematics, computer science, and physics.

Interestingly, ecology was included as “Environmental Sciences Ecology” in a middle ground among “The first 100 Research Areas” with 3,887 (0.82%) results, roughly one‐third from the biological field. Yet, the number of publications obtained through this filtering process still contained publications of several different areas. We therefore manually refined the selection of studies using X‐ray CT imaging in ecology, yielding a total of 81 (0.02% of total, but contributing 2.08% to the area of environmental sciences) studies retained in the final set (Appendix S1). A search for “Phase contrast tomography” and “Ecology” gave zero results.

**Figure 3:**
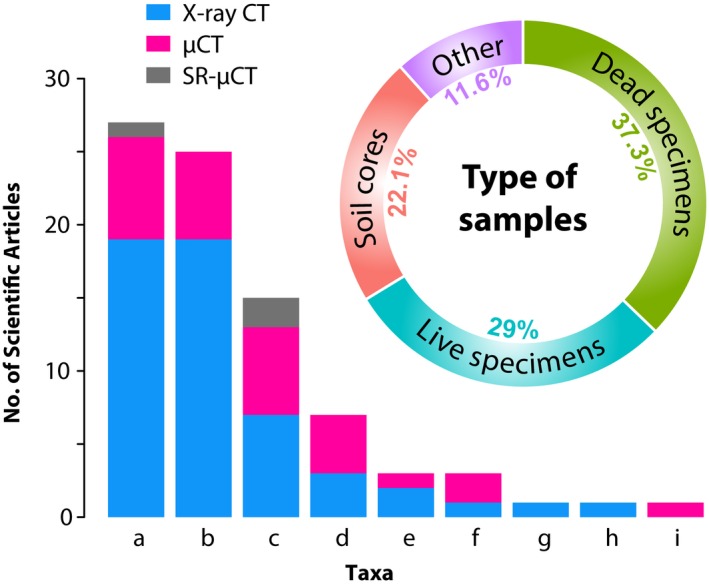
Quantitative analysis of ecological studies using x‐ray computed tomography. (a) Arthropoda, (b) Annelida, (c) Chordata, (d) Plantae (kingdom), (e) Cnidaria, (f) Mollusca, (g) Echinodermata, (h) Fungi (kingdom), (i) Porifera. μ‐CT: microcomputed tomography, SR‐μCT: synchrotron radiation microcomputed tomography

Comparing the total number of publications (476,232) with those presented in Appendix S1 (81) already shows how underexplored X‐ray CT in animal and—even more in—plant ecology is. From the scarce examples of ecological studies, the most studied animals have been arthropods and annelids, and in a lesser extend other kingdoms such as plants and fungi (Figure 3). It is particularly evident that one of the most popular topics so far is soil ecology (Davey et al., 2011; Harrison, Gardner, Tollner, & Kinard, 1993; Tollner, 1991), and specifically the study of worm burrows (Amossé, Turberg, Kohler‐Milleret, Gobat, & Le Bayon, 2015; Auclerc, Capowiez, Guérold, & Nahmani, 2013; Capowiez, Monestiez, & Belzunces, 2001; Capowiez, Pierret, & Moran, 2003; Francis, Tabley, Butler, & Fraser, 2001; Jégou, Capowiez, & Cluzeau, 2001; Jégou, Cluzeau, Hallaire, Balesdent, & Tréhen, 2000; Jégou, Cluzeau, Wolf, Gandon, & Tréhen, 1998; Jégou, Hallaire, Cluzeau, & Tréhen, 1999; Jégou et al., 2002; Langmaack, Schrader, Rapp‐Bernhardt, & Kotzke, 1999; Pagenkemper et al., 2015; Pelosi, Grandeau, & Capowiez, 2017; Rogasik, Schrader, Onasch, Kiesel, & Gerke, 2014; Schrader, Rogasik, Onasch, & Jegou, 2007), mostly because of the ease of studying this type of sample. Soil can be considered a matrix where the components can be detected through X‐ray CT without any preparation (i.e., staining). In soil samples, air spaces such as pores or worm burrows can be easily distinguished and measured. Additionally, invertebrates can be tracked in soil cores without requiring particular sample preparation due to their lower density compared to soil components (Tollner, 1991).

Another field with considerable usage of X‐ray CT is the study of social insects and their nest or gallery systems. Here, images are primarily acquired *in vivo* allowing to scan the same nest or colony several times during its developmental cycle (Eyer, Neumann, & Dietemann, 2016; Greco, Bell, Spooner‐Hart, & Holford, 2006; Greco, Spooner‐Hart, Beattie, Barchia, & Holford, 2011; Greco, Spooner‐Hart, & Holford, 2005; Rademacher, Fahlberg, Raddatz, Schneider, & Voigt, 2013). X‐ray CT has also proven to be a valuable tool for assessing processes in samples that do not allow direct visual evaluation without disturbing the organisms, such as parasitic relationships (Diez, Orensanz, Márquez, & Cremonte, 2013; Schwabe, Holtheuer, & Schories, 2014), seed‐feeding insects (Tarver et al., 2006), and growth strategies of animals (Cantin, Cohen, Karnauskas, Tarrant, & McCorkle, 2010; Fujiwara, Oji, Tanaka, & Kondo, 2005; Schönberg, 2001; Silbiger, Guadayol, Thomas, & Donahue, 2016), fungi (Van den Bulcke, Boone, Van Acker, & Van Hoorebeke, 2009), and plants (Dhondt et al., 2010; Ferreira et al., 2010; Gregory et al., 2003; Mairhofer et al., 2012; Mooney, Morris, & Berry, 2006; Perret, Al‐Belushi, & Deadman, 2007).

Establishment of trophic relationships in extant (Herrel et al., 2010; Kato et al., 2014; Pampush et al., 2016; Renaud et al., 2015; Self, 2015; Soons et al., 2015) and extinct species (Collareta et al., 2015; Gill et al., 2014) has also been possible by analysis of gut content, beak shape, or dental wear patterns.

In spite of the diversity of the topics explored in the ecological studies herein presented, there are scant examples of internal changes assessments, which in our opinion is one of the most promising avenues of the usage of X‐ray CT. Only some recent studies have shown changes in reproductive organs after mating and egg development in fruit flies (Mattei et al., 2015), and changes in volume of several organs of the trout after exposure to contaminated sediments (Brinkmann et al., 2016).

It is also interesting to note that, in terms of ecological applications, common X‐ray CT has been more widely used than μ‐CT (Figure 3). X‐ray CT scanners enable imaging of big samples (e.g., soil cores of 20 cm diameter and 70 height, pixel size around 200–400 μm) (Amossé et al., 2015; Pagenkemper et al., 2015), and although the rather low resolution allows accurate localization of internal structures in the nests of social insects (Fuchs, Schreyer, Feuerbach, & Korb, 2004; Greco et al., 2005, 2006), the identification of particular specimens is usually limited (Fuchs et al., 2004).

When scans of a single specimen are desired, resolutions of about 10 μm can be achieved during *in vivo* scanning (Postnov, De Clerck, Sasov, & Van Dyck, 2002; Yao et al., 2016). It is thus possible to reconstruct virtual cross sections through the specimens, even if respiratory, digestive, and/or cardiac systems are moving (De Clerck et al., 2004). However, volumetric assessments of particular organs *in vivo* have to be performed carefully as this technique can detect changes through time (e.g., several seconds) (Postnov et al., 2002; Westneat et al., 2008), which could cause measurement errors caused by variation in volume of structures due to normal physiological functions, such as ventilation and digestion. On the other hand, techniques for study real‐time dynamics (termed cine‐tomography) are being made available for 4D analysis (Rolo, Ershov, van de Kamp, & Baumbach, 2014).

When scanning live specimens, it is possible that organisms are negatively affected as a consequence of the absorbed radiation or overheating (Postnov et al., 2002). Depending on the resolution, area, and desired quality (signal‐to‐noise ratio), a scan might take from several minutes to hours (Dhondt et al., 2010). Several authors have claimed that repeated scans do not affect organisms (Postnov et al., 2002); in some studies, the same live specimens were scanned up to eight times (Halley, Burd, & Wells, 2005). When using live animals, the authors either did not assess possible side effects (Brinkmann et al., 2016) or just checked for a few hours or days after exposure (Dhondt et al., 2010).

In order to include the X‐ray CT analysis *in vivo* in ecological research, it is crucial to understand the effects of the radiation on the organisms before biological interpretation (Socha et al., 2007). In addition to the radiation, there are several steps of specimen preparation (such as staining and anesthesia) than can be potentially harmful. In some studies, tissue differentiation was achieved by staining live animals through dietary supplementation (e.g., with cadmium tungstate (CdWO_4_) and iodine) (Kim, Seo, Lim, & Lee, 2012; Socha et al., 2007), or injecting compounds into the circulatory system (Greco, Tong, Soleimani, Bell, & Schäfer, 2012). There are few studies about long‐term effects of X‐rays doses in invertebrates, and Kanao, Okamoto, Miyachi, and Nohara (2003) showed that low doses (0.5 Grays) caused transgenerational changes of emergence patterns in *Drosophila melanogaster* and this area would clearly need further study.

## 5 PREPARATION OF SAMPLES FOR X‐RAY CT

The preparation process preceding X‐ray CT scans is considerably shorter in comparison with classical histological techniques (Figure 1), in which obtaining a serial section of an average‐sized insect specimen (ca. 1 cm) can take several weeks (Friedrich & Beutel, 2008; Socha et al., 2007). In this review, we focus on the preparation of *ex vivo* specimens. This procedure usually comprises three simple steps: fixation, staining, and drying.

### 5.1 Fixation

Samples can be commonly fixed in ethanol or even embedded in resin (as in histology) (Metscher, 2013). For small vertebrates (e.g., laboratory mice, zebra fish, and embryos), it is also common to store specimen in formalin (Cnudde et al., 2008; Hoshino et al., 2012; Kim et al., 2012; Metscher, 2009a,b; Seo et al., 2015). Sombke et al. (2015) reported that fixation of several arthropod taxa in Bouin's solution provided better results in terms of tissue contrast when compared with ethanol and glutaraldehyde solution. To acquire images over short durations (e.g., to study mating), the sample can be flash‐frozen in liquid nitrogen (Mattei et al., 2015; Mouginot et al., 2015) and transferred to fixative solution afterward.

### 5.2 Staining

For soft tissues, X‐ray contrast can be enhanced using metal‐based stains, for instance, osmium tetroxide—widely used in transmission electron microscopy—and iodine. On the other hand, some structures can possess sufficient inherent contrast and do not require preparation or staining; this applies mainly to mineralized tissues such as bones or shells (Degenhardt, Wright, Horng, Padmanabhan, & Epstein, 2010; Metscher, 2013; Westneat et al., 2008). Proper differentiation of tissues within the sample is necessary for software‐based (semi‐) automatic recognition using thresholds of gray values (Friedrich & Beutel, 2008) or even for manually defining areas in each slice (Self, 2015). Metscher (2009a) suggested that stains are usually not tissue‐specific and the final quality depended mainly on the fixative. However, comparisons between different stains have shown that indeed some compounds can stain lipidic tissue more intensely (e.g., Lugol's solution) (Degenhardt et al., 2010); thus, recognition of different tissues can be greatly influenced by the chosen stain (Smith et al., 2016).

### 5.3 Drying

Samples can either be scanned in ethanol (inside plastic tubes that shall not interfere with the scanning process; plastic straws have proven to be good enough for this purpose) or dried and mounted/glued in custom‐made supports. Critical‐point drying (CPD, dehydration technique where water in biological tissue is replaced with CO_2_) gives good results preserving the fine structure of the sample (Beutel et al., 2014), keeping a high signal‐to‐noise ratio in the resulting images (Sombke et al., 2015). Chemical drying (e.g., using hexamethyldisilazane) is not recommendable because it causes tissue shrinkage and damage (e.g., in the brain of insects) (Sombke et al., 2015). At least air drying should be carried out to avoid shrinkage artifacts caused by water loss during the scanning process (Sena et al., 2015).

## 6 EXPERIMENTAL COMPARISON OF STAINING AND FIXATION APPROACHES

Although several combinations of methods for fixation, staining, and drying have previously been assessed and compared (Metscher, 2009a,b; Sombke et al., 2015), the authors generally used several species (vertebrate and invertebrate) and target tissues, and have carried out tomography without any phase retrieval. Here, we systematically compare different staining and fixation approaches, focusing on only one species, and consider in particular the more recent phase‐contrast modality. We combine techniques that previously proved successful, and provide results and comments about specific combinations in light of the desired results. As internal organs possess different densities (a very important characteristic when it comes to X‐ray‐based tomography) and chemical constitution (Nagy, 2001; Sterner & Elser, 2002), it is likely that one single perfect formulation is not possible in all cases. Here, we compare different methods to visualize structures in the head (mainly brain and muscles) and abdomen (ovaries and fat body) of *Acheta domesticus* (Linnaeus, 1758).

Commercially available *A. domesticus* adult females were euthanized in 70/30 solution ethanol/deionized water (although the specimens can also be fumigated with ethyl acetate or frozen as in Iwan, Kamimski, & Ras, 2015). Wings, legs, and antennae were removed with sharp dissecting scissors and discarded. The heads were removed from the bodies for separate scans, allowing better penetration of chemical compounds. For fixation, we used either ethanol (70/30 solution ethanol/deionized water), FAE (formaldehyde, acetic acid, and ethanol) or Bouin's solution (saturated aqueous picric acid, pure acetic acid, and formaldehyde), for 24 hr, following Beutel et al. (2014). For staining, either phosphotungstic acid (PTA) or iodine was used for 7 days and 24 hr, respectively, as described by Metscher (2009b). Every sample (e.g., head or abdomen) was scanned individually in random order either dry (air dried or critical‐point dried) or in ethanol. Every combination of fixative and staining was replicated three times (see Appendix S2 for detailed information about specimens’ preparation).

The samples (*N* = 48, Appendix S2) were scanned at the Institute for X‐ray Physics, University of Göttingen (Göttingen, Germany) using phase‐contrast μ‐CT techniques (for detailed specifications of the setup see Töpperwien, Krenkel, et al., 2016; Töpperwien, Krenkel, Quade, et al., 2016). Heads and abdomens were scanned using different detectors for a final pixel size effect of 2.6–2.7 (fiber‐coupled scintillator‐based sCMOS, Hamamatsu Photonics, Japan) and 10 μm (Dexela CMOS Flat Panel, PerkinElmer, Germany), respectively. In both cases, we acquired 1,000 projections in an angular range of 183–190°. After raw image correction (dark current subtraction and empty beam division), the projection images, which exhibited the typical edge enhancement as a manifestation of phase contrast, were treated using the Bronnikov‐aided correction (BAC) algorithm proposed by De Witte, Boone, Vlassenbroeck, Dierick, and Van Hoorebeke (2009). A phase retrieval assuming vanishing absorption was carried out based on Fourier filtering, followed by a correction step to represent the amplitude of the exit wave. As explained in Töpperwien, Krenkel, Quade, et al. (2016), Töpperwien et al. (2017), the BAC scheme inverts blur by diffraction, achieves higher sharpness in comparison with other schemes, while partially mixing amplitude and phase contrast to an effective contrast. The details of the data analysis used here closely follow the procedures published before (Töpperwien, Krenkel, Quade, et al. (2016); Töpperwien et al. (2017)).

**Figure 4:**
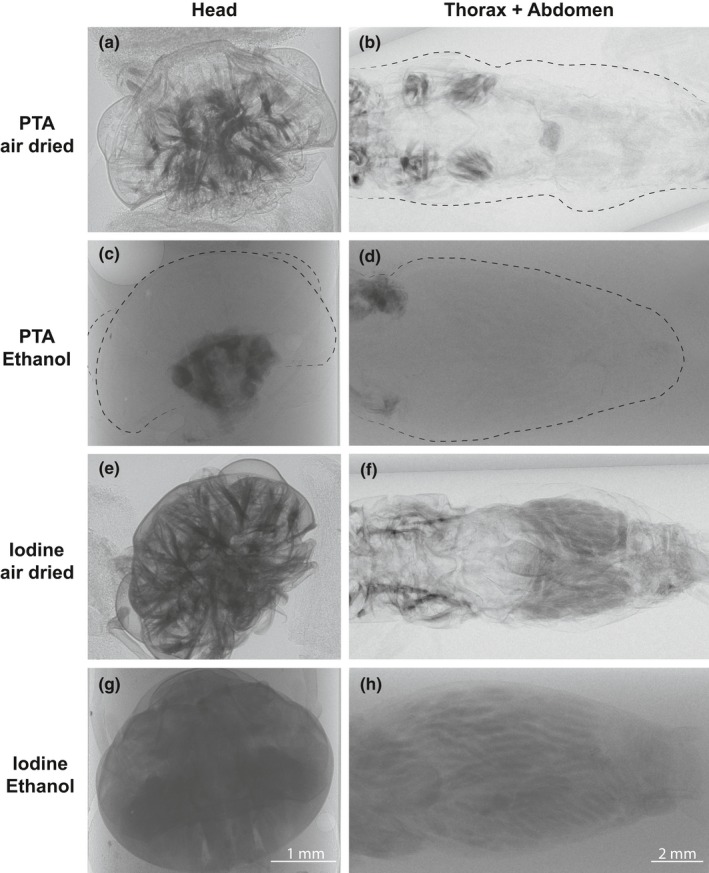
Experimental comparison of staining agents and scanning mediums using only ethanol as fixative in the cricket *Acheta domesticus*. After fixation for 24 hr in 70/30 solution ethanol/deionized water, samples were stained in phosphotungstic acid (PTA) or iodine (solution with ethanol) during 7 days and 24 hr, respectively. Posteriorly, the samples were scanned either in ethanol or air‐dried

Iodine proved to be the best staining agent in our tests, because of its faster tissue penetration and superior overall contrast across all tissues (Figure 4e‐h), also the usage of low concentrations assured minimum artifacts (Vickerton, Jarvis, & Jeffery, 2013). PTA failed to stain the complete sample (neither head nor abdomen), and the few stained regions had very strong contrast, precluding adequate tissue recognition (Figure 4a‐d). The unstained parts of the samples exposed to PTA had minor contrast, resembling tissues that had not been exposed to any stain (Degenhardt et al., 2010; Metscher, 2009a), thus indicating that the tissue penetration was insufficient and samples would have to be exposed for more days to attain the desired stain.

PTA has shown to yield sufficient results to recognize of brain and muscle structures in arthropods (Smith et al., 2016; Swart, Wicklein, Sykes, Ahmed, & Krapp, 2016). However, because of its slow penetration rate—as evidenced by Smith et al. (2016) and also this study—it might be considered when the sample tissue is thin or removal of parts of the exoskeleton is possible (to facilitate stain perfusion). While osmium tetroxide (OsO_4_) has been a popular staining agent in previous X‐ray CT studies (Jahn et al., 2018; Kim et al., 2012; Metscher, 2009a,b; Ribi, Senden, Sakellariou, Limaye, & Zhang, 2008), we did not consider it because of its undesirable toxicity, high costs, limited tissue penetration, and failure to work properly in tissues preserved in alcohol (Hayat, 1970; Metscher, 2009a; Smith, Carson, & Ferguson, 1974; Smith et al., 2016).

With respect to the scanning medium, dried samples (air drying technique for this test) provided the best results. Many of the structures are already evident in projections prior to reconstruction (Figure 2e, f). Using ethanol as medium could possibly prevent artifacts (e.g., tissue shrinkage), but the contrast was greatly reduced and scanning time had to be tripled.

**Figure 5:**
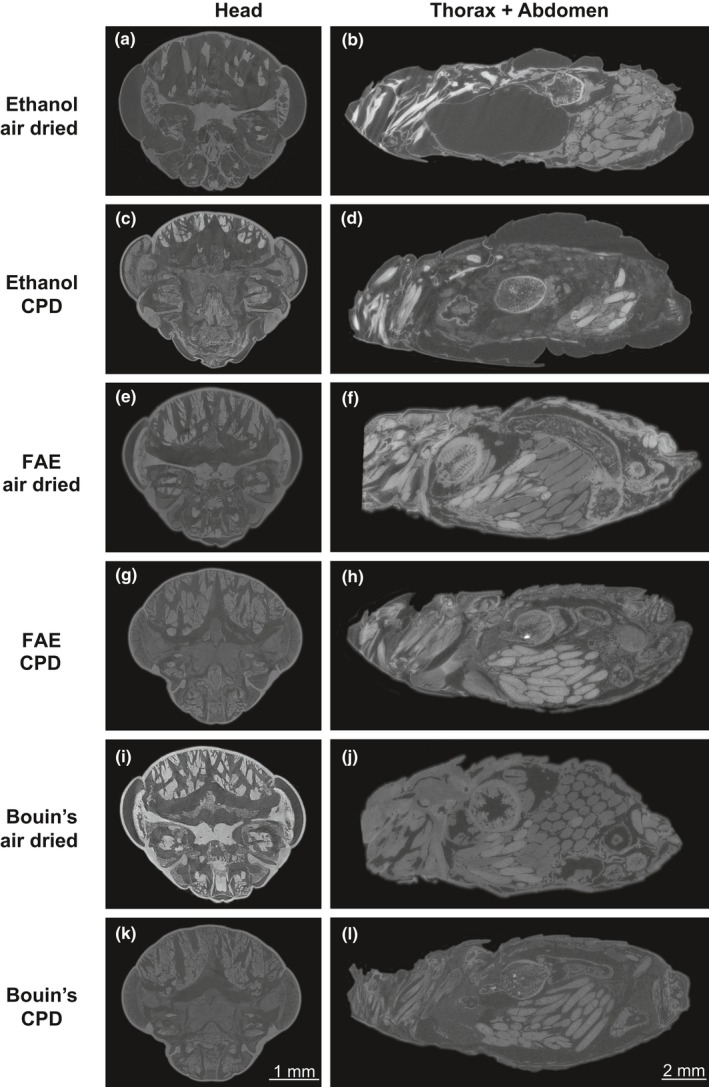
Experimental comparison of fixatives and drying techniques using iodine as chosen staining agent in the cricket *Acheta domesticus*. After fixation for 24 in either 70/30 solution ethanol/deionized water, FAE (formaldehyde, acetic acid, and ethanol), or Bouin's solution (saturated aqueous picric acid, pure acetic acid, and 10% formaldehyde solution), the samples were air‐dried or submitted to CPD (critical‐point drying) before being scanned. Heads are presented in coronal planes and abdomens in sagittal planes

After having found an appropriate staining agent and scanning medium, we proceeded to test how the selection of the fixative and the drying technique affected the final result. Ethanol (as a fixative) provided undesirable results due to strong artifacts (e.g., air spaces and tissue shrinkage) especially when combined with air drying (Figure 5a, b). Although ethanol with CPD provided sufficient contrast, separation of tissue from the cuticle was still evident (Figure 5c, d). Samples fixed with Bouin's and air dried appeared overstained and also showed artifacts (Figure 5i, j). However, the usage of Bouin's in combination with CPD showed better results (Figure 5k, l). FAE fixative provided good contrast and quality in general when either air‐ or critical‐point dried; tissue conformation appeared natural and with few artifacts.

As expected, regardless of the fixative used, CPD significantly increased stain complexity and quality (e.g., brain lobes in Figure 5g, h). This drying technique also avoided overstaining in combination Bouin's iodine. When brain and muscle morphology is the objective of the study, the sample should be critical‐point dried after fixation (with either FAE or Bouin's) and staining (iodine). If other tissues, such as ovaries, gut, or fat body, are targeted, FAE fixation should be prioritized and air drying provides good results. Air drying reduces the cost and time for sample preparation prior scanning.

### 6.1 Sample size, effort, and costs

In studies intending to include X‐ray CT imaging, the number of samples to be analyzed (i.e., prepared, scanned, reconstructed, and segmented) would depend to a large extent on the time necessary from obtaining the specimen/sample until data collection, and the costs associated with this process.

From several publications included in our historic review (Appendix S1, Figure 2), it is clear that sample size tended to be limited so far. For instance, Brinkmann et al. (2016) used just three specimens (rainbow trout) per treatment, although with a laborious segmentation process. Further, Greco et al. (2010) used five stingless‐bee hives to study the defensive mechanism against parasite beetles. On the other hand, when samples (e.g., soil cores) or specimens used allowed for an easier segmentation (because of higher contrast and therefore possible semiautomatic recognition), sample size tended to be bigger (*N* = 8–20, with more than 50 scans per study) (Himmi et al., 2016; Monaenkova et al., 2015; Pelosi et al., 2017; Silbiger et al., 2016).

Additionally, the sample size can be increased by batch‐preparing (fixation, staining, drying) and scanning the samples. Several specimens can be scanned at the same time as in Smith et al. (2016) and separated for individual analysis in the segmentation process.

On the other hand, the nature of the samples or specimens can also limit sample sizes in ecological studies. In some cases (e.g., fossils or rare species), few samples or specimens are available for analysis but the results are not less significant (Collareta et al., 2015; Gill et al., 2014).

With more manufacturers (Appendix S3) and institutions with facilities dedicated to X‐ray CT imaging, the cost of devices and scanning services will continue to decrease and this technology will become more accessible in the near future. Comparisons in terms of costs in the utilization or decision between techniques have been discussed previously (Cunningham, Rahman, Lautenschlager, Rayfield, & Donoghue, 2014). Recently, Silbiger et al. (2016) reported costs as low as $100 per scan.

Regarding the effort required to fully processes the samples, besides batch‐preparing, an optimization of the protocol for the model organism (or specific type of sample) is highly recommended. Pilot scans of the focal tissue pursuing an optimal fixation and staining time would enhance contrast, thus easing the segmentation process.

Individual researchers performing the segmentations have to undergo a training phase in order to reduce human‐introduced errors. After this phase, the segmentation and subsequent calculation of volumes can be performed in a short period of time (usually few hours when semiautomatic segmentation is possible).

The procedure used for specimen preparation in the present study is straightforward and can be carried out in any laboratory as we gave preference to nontoxic chemicals. Once the desired specimen is ready for preparation, it has to be fixated (2–12 hr depending on size), stained (24 hr when using iodine) and dried (approx. 1 hr for CPD). Time necessary to scan a sample was about 8.33 min (1,000 projections at an angular range of 190°, and 0.5 s of exposure time). The reconstruction process can be completed in ca. 20 min running the script in MATLAB (this last step is naturally dependent on computing power). After obtaining the final reconstructed file, this can be loaded in the software for segmentation (in this case, we used Seg3D) and the thresholding and cleaning of the region of interest (i.e., ovaries) took in average two hours per file. Overall, we estimate the current time budget needed per sample to be around 5 hr excluding fixation. As many processes can now be run on entire batches of samples, this is not more time‐consuming that other imaging techniques such as electron microscopy or fluorescence microscopy.

## 7 PROCESSING OF X‐RAY CT IMAGES

Once the samples are scanned, and the resulting projections (as in Figure 4) have undergone the phase retrieval step described above, tomographic reconstruction is carried out in order to obtain the slices (as presented in Figure 5). For instance, from 1,000 projections of a cricket head, 2,048 slices were acquired after reconstruction. Several algorithms are available for this task, for a thorough explanation of this process we encourage the reader to consult the study by Willemink et al. (2013). Here, raw data corrections and phase retrieval of projections were carried out in MATLAB (The MathWorks, Inc., Massachusetts, United States), and cone‐beam reconstruction was carried out using the ASTRA toolbox (van Aarle et al., 2016) interfaced with MATLAB.

The slices obtained after the reconstruction can be analyzed in many ways. For instance, inspection of tissue conformation in any anatomical plan, description of organs, and two‐dimensional measurements can be performed in any software for image visualization such as ImageJ (Schneider, Rasband, & Eliceiri, 2012). However, three‐dimensional measurements (i.e., volumes) are the most desired feature of X‐ray CT. In order to measure a single organ or structure contained in our scan, this has to be segmented; that is, the pixels associated with the structure have to be selected and labeled.

Segmentation is one of the most important steps in X‐ray CT data processing, and perhaps the most labor‐intensive part depending on the desired data. Several authors have compared the capabilities of the available software (Lautenschlager, 2016) and step‐by‐step protocols have been published recently (Abel, Laurini, & Richter, 2012; Fedorov et al., 2012; Smith et al., 2016; Supporting information), but still segmentation remains largely context‐dependent (Swart, Deaton, & Felgenhauer, 2006).

This process can be performed using commercially available (e.g., Avizo, AVGStudio), open‐source software (e.g., SPIERS, Seg3D, 3D Slicer) (Fedorov et al., 2012; Sutton, Garwood, Siveter, & Siveter, 2012; Tate, Burton, & Khan, 2016), and online applications (e.g., Biomedisa, Lösel & Heuveline, 2016). In addition to 3D reconstruction, some of these available software packages offer the possibility to measure linear features, areas, and volumes as well (Ravel & Orliac, 2015). Commonly, data (e.g., volumes) are obtained in voxels (from the contraction of *vox* “volume” and *el* for “element”), which is a unit of graphic information that defines a point in three‐dimensional space, each coordinate being defined in terms of its position, color, and density (Higgins, Williams, Nagel, & Higgins, 2006). The volume of the structure of tissue can be calculated through the voxel size (e.g., in this study, the voxel size for the cricket head was 2.7 μm^3^).

Several authors have pointed out how difficult the segmentation process can be (Greco et al., 2012; Gremse et al., 2015; Kim et al., 2012), mainly due to poorly contrasted regions (in many cases resulting from inadequate sample preparation). Although present‐day computers allow fast and accurate processing of tomographic data (Mattei et al., 2015), Metscher (2013) claimed that the development of novel machine‐learning algorithms operating in three‐dimensional space will be needed for 3D segmentation and semiautomatic comparisons of images (e.g., variation and growth of structures). Fast recognition of regions in the volumes would reduce the time and increase the accuracy of the quantitative analysis of a large number of datasets, thus allowing for more robust statistics (Sickert et al., 2015; Smith et al., 2016).

**Figure 6:**
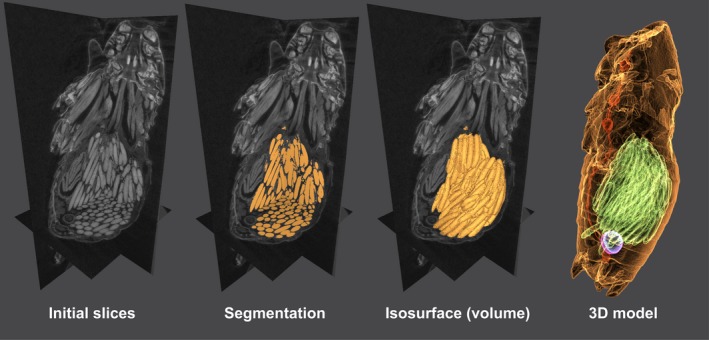
Segmentation process steps. *Acheta domesticus* female abdomen. Please click on the figure to activate the interactive 3D content and use the mouse to rotate the objects. Further functions (views, render modes, and model tree) are available in the menu

From our house crickets’ dataset, we chose the ovaries and spermathecae as focal tissues due to their high contrast in comparison with the surrounding tissues. By way of example, the segmentation was performed semiautomatically using Seg3D (Tate et al., 2016) from a single scan of an abdomen fixed with FAE, stained with iodine and dried using CDP (Figure 6). First, the slices were subjected to thresholding until the desired region was completely highlighted. Subsequently, a crop mask was applied to select the region of interest, and finally, undesired highlighted areas (belonging to other tissues) were erased manually using the brush tool. In the interactive Figure 6, the segmented ovaries (4.02e^7^ voxels, 0.398 mm^3^), spermatheca (1.44e^6^ voxels, 0.014 mm^3^), and stored sperm (1.04e^6^ voxels, 0.010 mm^3^) are presented. The ventral nervous cord (8.16e5 voxels, 8.08e^−3^ mm^3^), also included in Figure 6, is presented as an example of a manually segmented region (its low contrast precluded semiautomatic segmentation).

Care has to be taken when segmenting several specimens for quantitative purposes as variations as high as 20% have been reported (Parkinson, Badiei, & Fazzalari, 2008), however, using proper tissue preparation (staining and drying) and scanning techniques (e.g., phase contrast) this variation can be reduced. Besides, techniques for calibration are been developed, which would greatly increase the accuracy of the measurements (Léonard, Brown, Withers, Mummery, & McCarthy, 2014).

Due to the considerable size of the reconstructed scans (several gigabytes in most cases), it is usually not possible to include these as Supporting information to manuscripts. However, long‐lasting online repositories such as Digital Morphology Library (http://www.digimorph.org), Dryad Digital Repository (http://datadryad.org), MorphoBank (https://morphobank.org), MorphoSource (http://morphosource.org), and Science3D (https://www.science3d.org) provide storage facilities to ease the publication of datasets and accessibility to the scanned specimens. Outstanding examples of this possibility are the cybertypes of recently described species (Akkari et al., 2015) and online repositories of fossils (UMORF, https://umorf.ummp.lsa.umich.edu/).

## 8 PERSPECTIVES AND FINAL CONSIDERATIONS

As pointed out before in several reviews (Metscher, 2013; Socha et al., 2007; Westneat et al., 2008), X‐ray CT bears the potential to generate valuable morphological data from specimens and structures that would be impossible or expensive to acquire using other approaches (e.g., synchrotron or MRI facilities) (Brinkmann et al., 2016) or that would be time‐consuming (e.g., microtomy). Although reconstruction artifacts will inevitably be present in some images (Davis & Elliott, 2006), in this review we present several successful applications of this technique—and its variations—in ecological studies (Appendix S1). Our study outlines suitable combinations of techniques for preparation of specimen, providing sufficient contrast for image segmentation.

To date, X‐ray CT has proven to be a suitable technique to reveal the biology and ecology of elusive organisms (Jennings & Austin, 2011; Mouginot et al., 2015), diet analysis of extinct species (Collareta et al., 2015; Gill et al., 2014), and to examine nest/colony life, development, and structure (Fuchs et al., 2004; Greco et al., 2005, 2006). In terms of applied studies, monitoring of pest and invasive species (Fuchs et al., 2004; Halley et al., 2005; Harrison et al., 1993; Monaenkova et al., 2015; Soné, Mori, Ide, Setoguchi, & Yamanouchi, 1995; Tarver et al., 2006), interaction between hosts and parasites (Greco et al., 2010; Schwabe et al., 2014), forensic entomology (Johnson et al., 2012), and ecotoxicological research (Brinkmann et al., 2016; Holliday & Holliday, 2012; Lind et al., 2004; Pigneret et al., 2016; Yunusa, Braun, & Lawrie, 2009) have been also benefited greatly from this technique so far.

As the tissue detection in the technique explored in this review relies on metal‐based stains, X‐ray CT can also be considered as a method to detect, monitor, and even infer physiological pathways of metallic pollution in small animals (Bell et al., 2012). Furthermore, combination with other techniques—for instance fluorescence microscopy—may allow to identify processes within the tissues for future studies on ecophysiology and ecotoxicology (e.g., angiogenesis, apoptosis, inflammation) (Gremse et al., 2015; Handschuh, Baeumler, Schwaha, & Ruthensteiner, 2013; Metscher, 2009a). Possible detrimental effects of radiation and stain ingestion on living organisms (especially invertebrates) remain to be elucidated.

On the other hand, metal‐based staining is not a prerequisite, as evidence by the ethanol fixated and air‐dried sample (Figure 5a, b). Similar to synchrotron radiation, where the signal‐to‐noise is high enough to detect anatomical and histological details in unstained specimens, and similar to previous studies in other biological samples (Bartels et al., 2013; Krenkel et al., 2015; Töpperwien, Krenkel, Quade, et al., 2016), suitable drying procedures may thus be sufficient to yield sufficient image contrast also for insects and arthropods.

Overall, X‐ray computed tomography bears tremendous potential for future ecological research. We are just starting to unravel these possibilities.

## CONFLICT OF INTEREST

None declared.

## AUTHORS’ CONTRIBUTIONS

CS, YG, and DO conceived the idea of the manuscript. YG, TS, and MT designed the methodology and performed the scans. YG collected the literature data and led the writing of the manuscript. All authors gave final approval for publication.

## DATA ACCESSIBILITY

Detailed information about the studies included in the historical review (Appendix S1), details of scans and specimens’ preparation (Appendix S2), updated list of manufacturers (Appendix S3), and a video of the scans used for Figure 6 (Video S4) can be found as online Supporting information. Representative scans (reconstructed files) of the techniques compared in this study are available from the Dryad Digital Repository: https://doi.org/10.5061/dryad.f9h43b0.
