# Supplementary material for: X‐ray computed tomography and its potential in ecological research: A review of studies and optimization of specimen preparation
Source: Ecol Evol. 2018 Jul 6;8(15):7717–32. doi: 10.1002/ece3.4149 (PMC6106166; doi:10.1002/ece3.4149)
Supplement: Supplementary file 1 [file ECE3-8-7717-s001.pdf]

Amossé, J., Turberg, P., Kohler-Milleret, R., Gobat, J.M. & Le Bayon, R.C. (2015) Effects of endogeic earthworms on the soil organic matter dynamics and the soil structure in urban and alluvial soil materials. *Geoderma*, **243–244**, 50–57.

Auclerc, A., Capowiez, Y., Guérold, F. & Nahmani, J. (2013) Application of X-ray tomography to evaluate liming impact on earthworm burrowing activity in an acidic forest soil under laboratory conditions. *Geoderma*, **202–203**, 45–50.

Brinkmann, M., Rizzo, L.Y., Lammers, T., Gremse, F., Schiwy, S., Kiessling, F. & Hollert, H. (2016) Micro-computed tomography (μCT) as a novel method in ecotoxicology - determination of morphometric and somatic data in rainbow trout (Oncorhynchus mykiss). *Science of the Total Environment*, **543**, 135–139.

Van den Bulcke, J., Boone, M., Van Acker, J. & Van Hoorebeke, L. (2009) Three-dimensional x-ray imaging and analysis of fungi on and in wood. *Microscopy and microanalysis : the official journal of Microscopy Society of America, Microbeam Analysis Society, Microscopical Society of Canada*, **15**, 395–402.

Cantin, N.E., Cohen, A.L., Karnauskas, K.B., Tarrant, A.M. & McCorkle, D.C. (2010) Ocean Warming Slows Coral Growth in the Central Red Sea. *Science*, **329**, 322–325.

Capowiez, Y., Monestiez, P. & Belzunces, L. (2001) Burrow systems made by Aporrectodea nocturna and Allolobophora chlorotica in artificial cores: Morphological differences and effects of interspecific interactions. *Applied Soil Ecology*, **16**, 109–120.

Capowiez, Y., Pierret, A. & Moran, C.J. (2003) Characterisation of the three-dimensional structure of earthworm burrow systems using image analysis and mathematical morphology. *Biology and Fertility of Soils*, **38**, 301–310.

Collareta, A., Landini, W., Lambert, O., Post, K., Tinelli, C., Celma, C. Di, Panetta, D., Tripodi, M., Salvadori, P.A., Caramella, D., Marchi, D., Urbina, M. & Bianucci, G. (2015) Piscivory in a Miocene Cetotheriidae of Peru: first record of fossilized stomach content for an extinct baleen-bearing whale. *The Science of Nature*, **102**, 2–12.

Czachor, H., Charytanowicz, M., Gonet, S., Niewczas, J., Jozefaciuk, G. & Lichner, L. (2015) Impact of long-term mineral and organic fertilizer application on the water stability, wettability and porosity of aggregates obtained from two loamy soils. *European Journal of Soil Science*, **66**, 577–588.

Danner, R.M., Gulson-castillo, E.R., James, H.F., Dzielski, S.A., David, C., Iii, F., Sibbald, E.T., Winkler, D.W., Danner, R.M., Gulson-castillo, E.R., James, H.F., Dzielski, S.A., Iii, D.C.F., Sibbald, E.T. & Winkler, D.W. (2017) Habitat-specific divergence of air conditioning structures in bird bills. *The Auk*, **134**, 65–75.

Davey, E., Wigand, C., Johnson, R., Sundberg, K., Morris, J. & Roman, C.T. (2011) Use of computed tomography imaging for quantifying coarse roots, rhizomes, peat, and particle densities in marsh soils. *Ecological Applications*, **21**, 2156–2171.

Delefosse, M., Kristensen, E., Crunelle, D., Braad, P.E., Dam, J.H., Thisgaard, H., Thomassen, A. & Høilund-Carlsen, P.F. (2015) Seeing the unseen-bioturbation in 4d: Tracing bioirrigation in marine sediment using positron emission tomography and computed tomography. *PLoS ONE*, **10**, 1–17.

Dhondt, S., Vanhaeren, H., Van Loo, D., Cnudde, V. & Inzé, D. (2010) Plant structure visualization by high-resolution X-ray computed tomography. *Trends in Plant Science*, **15**, 419–422.

Diez, M.E., Orensanz, J.M., Márquez, F. & Cremonte, F. (2013) Shell damage in the Tehuelche scallop Aequipecten tehuelchus caused by Polydora rickettsi (Polychaeta: Spionidae) infestation. *Journal of Invertebrate Pathology,* **114**, 107–113.

El-Mallakh, R.S. (1993) Carpenter Ant (Hymenoptera - Formicidae) Tunnels Visualized By Computed tomography. *Entomological News*, **104**, 5.

Eyer, M., Neumann, P. & Dietemann, V. (2016) A Look into the Cell : Honey Storage in Honey Bees , Apis mellifera. *PLoS ONE*, **11**, 1–20.

Ferreira, S.J., Senning, M., Sonnewald, S., Kessling, P.-M., Goldstein, R. & Sonnewald, U. (2010) Comparative transcriptome analysis coupled to X-ray CT reveals sucrose supply and growth velocity as major determinants of potato tuber starch biosynthesis. *BMC genomics*, **11**, 93.

Francis, G.S., Tabley, F.J., Butler, R.C. & Fraser, P.M. (2001) The burrowing characteristics of three common earthworm species. *Soil Research*, **39**, 1453–1465.

Fuchs, A., Schreyer, A., Feuerbach, S. & Korb, J. (2004) A new technique for termite monitoring using computer tomography and endoscopy. *International Journal of Pest Management*, **50**, 63–66.

Fujiwara, S.-I., Oji, T., Tanaka, Y. & Kondo, Y. (2005) Relay strategy and adaptation to a muddy environment in Isselicrinus (Isselicrinidae: Crinoidea). *Palaios*, **20**, 241–248.

Gill, P.G., Purnell, M. a., Crumpton, N., Brown, K.R., Gostling, N.J., Stampanoni, M. & Rayfield, E.J. (2014) Dietary specializations and diversity in feeding ecology of the earliest stem mammals. *Nature,* **512**, 303–305.

Greco, M., Bell, M., Spooner-Hart, R. & Holford, P. (2006) X-ray computerized tomography as a new method for monitoring Amegilla holmesi nest structures, nesting behaviour, and adult female activity. *Entomologia Experimentalis et Applicata*, **120**, 71–76.

Greco, M.K., Hoffmann, D., Dollin, A., Duncan, M., Spooner-Hart, R. & Neumann, P. (2010) The alternative Pharaoh approach: Stingless bees mummify beetle parasites alive. *Naturwissenschaften*, **97**, 319–323.

Greco, M.K., Lang, J., Gallmann, P., Priest, N., Feil, E. & Crailsheim, K. (2013) Sugar concentration influences decision making in *Apis mellifera* L. workers during early-stage honey storage behaviour. *Open Journal of Animal Sciences*, **3**, 210–218.

Greco, M. & Sadd, B. (2012) Using diagnostic radioentomology for non-invasive observations of colonies of the bumblebee, Bombus terrestris. *Journal of Insect Science*, **12**, 1–6.

Greco, M.K., Spooner-Hart, R.N., Beattie, A.G. a C., Barchia, I. & Holford, P. (2011) Australian stingless bees improve greenhouse Capsicum production. *Journal of Apicultural Research*, **50**, 102–115.

Greco, M., Spooner-Hart, R. & Holford, P. (2005) A new technique for monitoring Trigona carbonaria nest contents , brood and activity using X-ray computerized tomography. *Journal of Apicultural Research*, **44**, 97–100.

Greco, M.K., Tong, J., Soleimani, M., Bell, D. & Schäfer, M.O. (2012) Imaging live bee brains using minimally-invasive diagnostic radioentomology. *Journal of insect science (Online),* **12**, 1–7.

Gregory, P.J., Hutchison, D.J., Read, D.B., Jenneson, P.M., Gilboy, W.B. & Morton, E.J. (2003) Non-invasive imaging of roots with high resolution X-ray micro-tomography.

Plant and Soil, **255**, 351–359.

Halley, J.D., Burd, M. & Wells, P. (2005) Excavation and architecture of Argentine ant nests. *Insectes Sociaux*, **52**, 350–356.

Harrison, R.D., Gardner, W.A., Tollner, W.E. & Kinard, D.J. (1993) X-ray Computed Tomography Studies of the Burrowing Behavior of Fourth-Instar Pecan Weevil (Coleoptera: Curculionidae). *Journal of Economic Entomology*, **86**, 1714–1719.

Hasumura, T. & Meguro, S. (2016) Exercise quantity‐dependent muscle hypertrophy in adult zebrafish (Danio rerio). *Journal of Comparative Physiology B*, **186**, 603–614.

Herrel, A., Soons, J., Aerts, P., Dirckx, J., Boone, M., Jacobs, P., Adriaens, D. & Podos, J. (2010) Adaptation and function of the bills of Darwins finches: Divergence by feeding type and sex. *Emu*, **110**, 39–47.

Higgs, N.D., Glover, A.G., Dahlgren, T.G. & Little, C.T.S. (2010) Using computed-tomography to document borings by Osedax mucofloris in whale bone. *Cahiers de Biologie Marine*, **51**, 401–405.

Higgs, N.D., Glover, A.G., Dahlgren, T.G. & LITTLE, C.T.S. (2011) Bone-boring worms: Characterizing the morphology, rate, and method of Bioerosion by Osedax mucofloris (Annelida, Siboglinidae). *Biological Bulletin*, **221**, 307–316.

Higgs, N.D., Glover, A.G., Dahlgren, T.G., Smith, C.R., Fujiwara, Y., Pradillon, F., Johnson, S.B., Vrijenhoek, R.C. & Little, C.T.S. (2014) The morphological diversity of Osedax worm borings (Annelida: Siboglinidae). *Journal of the Marine Biological Association of the United Kingdom*, **94**, 1429–1439.

Himmi, S.K., Yoshimura, T., Yanase, Y., Oya, M., Torigoe, T., Akada, M. & Imadzu, S. (2016) Nest-Gallery Development and Caste Composition of Isolated Foraging Groups of the Drywood Termite Incisitermes minor (Isoptera: Kalotermitidae). *Insects*, **7**, 2–14.

Holden, A.R., Koch, J.B., Griswold, T., Erwin, D.M. & Hall, J. (2014) Leafcutter bee nests and pupae from the Rancho La Brea Tar Pits of southern California: Implications for understanding the paleoenvironment of the Late Pleistocene. *PLoS ONE*, **9**, 1–12.

Holliday, D.K. & Holliday, C.M. (2012) The effects of the organopollutant PCB 126 on bone density in juvenile diamondback terrapins (Malaclemys terrapin). *Aquatic Toxicology*, **109**, 228–233.

Jeffery, S., Meinders, M.B.J., Stoof, C.R., Bezemer, T.M., van de Voorde, T.F.J., Mommer, L. & van Groenigen, J.W. (2015) Biochar application does not improve the soil hydrological function of a sandy soil. *Geoderma*, **251–252**, 47–54.

Jégou, D., Brunotte, J., Rogasik, H., Capowiez, Y., Diestel, H., Schrader, S. & Cluzeau, D. (2002) Impact of soil compaction on earthworm burrow systems using X-ray computed tomography: Preliminary study. *European Journal of Soil Biology*, **38**, 329–336.

Jégou, D., Capowiez, Y. & Cluzeau, D. (2001) Interactions between earthworm species in artificial soil cores assessed through the 3D reconstruction of the burrow systems. *Geoderma*, **102**, 123–137.

Jégou, D., Cluzeau, D., Hallaire, V., Balesdent, J. & Tréhen, P. (2000) Burrowing activity of the earthworms Lumbricus terrestris and Aporrectodea giardi and consequences on C transfers in soil. *European Journal of Soil Biology*, **36**, 27–34.

Jégou, D., Cluzeau, D., Wolf, H.J., Gandon, Y. & Tréhen, P. (1998) Assessment of the burrow system of Lumbricus terrestris, Aporrectodea giardi, and Aporrectodea caliginosa using X-ray computed tomography. *Biology and Fertility of Soils*, **26**, 116–121.

Jégou, D., Hallaire, V., Cluzeau, D. & Tréhen, P. (1999) Characterization of the burrow system of the earthworms Lumbricus terrestris and Aporrectodea giardi using X-ray computed tomography and image analysis. *Biology and Fertility of Soils*, **29**, 314–318.

Jennings, J.T. & Austin, A.D. (2011) Novel use of a micro-computed tomography scanner to trace larvae of wood boring insects. *Australian Journal of Entomology*, **50**, 160–163.

Johnson, A., Archer, M., Leigh-Shaw, L., Pais, M., O'Donnell, C. & Wallman, J. (2012) Examination of forensic entomology evidence using computed tomography scanning: Case studies and refinement of techniques for estimating maggot mass volumes in bodies. *International Journal of Legal Medicine*, **126**, 693–702.

Johnson, S.N., Read, D.B. & Gregory, P.J. (2004) Tracking larval insect movement within soil using high resolution X-ray microtomography. *Ecological Entomology*, **29**, 117–122.

Joschko, M., Graff, O., Müller, P.C., Kotzke, K., Lindner, P., Pretschner, D.P. & Larink, O. (1991) A non-destructive method for the morphological assessment of earthworm burrow systems in three dimensions by X-ray computed tomography. *Biology and Fertility of Soils*, **11**, 88–92.

Joschko, M., Müller, P.C., Kotzke, K., Döhring, W. & Larink, O. (1993) Earthworm burrow system development assessed by means of X-ray computed tomography. *Geoderma*, **56**, 209–221.

Kato, A., Tang, N., Borries, C., Papakyrikos, A.M., Hinde, K., Miller, E., Kunimatsu, Y., Hirasaki, E., Shimizu, D. & Smith, T.M. (2014) Intra- and interspecific variation in Macaque molar enamel thickness. *American Journal of Physical Anthropology*, **155**, 447–459.

Kiel, S., Kahl, W.A. & Goedert, J.L. (2013) Traces of the bone-eating annelid Osedax in Oligocene whale teeth and fish bones. *Palaontologische Zeitschrift*, **87**, 161–167.

Kim, B.H., Seo, E.S., Lim, J.H. & Lee, S.J. (2012) Synchrotron X-ray microscopic computed tomography of the pump system of a female mosquito. *Microscopy Research and Technique*, **75**, 1051–1058.

Laforsch, C., Christoph, E., Glaser, C., Naumann, M., Wild, C. & Niggl, W. (2008) A precise and non-destructive method to calculate the surface area in living scleractinian corals using X-ray computed tomography and 3D modeling. *Coral Reefs*, **27**, 811–820.

Langmaack, M., Schrader, S., Rapp-Bernhardt, U. & Kotzke, K. (1999) Quantitative analysis of earthworm burrow systems with respect to biological soil-structure regeneration after soil compaction. *Biology and Fertility of Soils*, **28**, 219–229.

Lind, P.M., Milnes, M.R., Lundberg, R., Bermudez, D., Örberg, J. & Guillette, L.J. (2004) Abnormal bone composition female juvenile American alligators from a pesticide-polluted lake (Lake Apopka, Florida). *Environmental Health Perspectives*, **112**, 359–362.

Mairhofer, S., Zappala, S., Pridmore, T., Mairhofer, S., Zappala, S., Tracy, S.R., Sturrock, C., Bennett, M., Mooney, S.J. & Pridmore, T. (2012) RooTrak : Automated Recovery of Three- Dimensional Plant Root Architecture in Soil from X-Ray Microcomputed Using Visual Tracking. *Plant Physiology*, **158**, 561–569.

Mattei, A.L., Riccio, M.L., Avila, F.W. & Wolfner, M.F. (2015) Integrated 3D view of postmating responses by the Drosophila melanogaster female reproductive tract, obtained by micro-computed tomography scanning. *Proceedings of the National Academy of Sciences of the United States of America*, **112**, 8475–8480.

Monaenkova, D., Gravish, N., Rodriguez, G., Kutner, R., Goodisman, M.A.D. & Goldman, D.I. (2015) Behavioral and mechanical determinants of collective subsurface nest excavation. *Journal of Experimental Biology*, **218**, 1295–1305.

Mooney, S.J., Morris, C. & Berry, P.M. (2006) Visualization and Quantification of the Effects of Cereal Root Lodging on Three-Dimensional Soil Macrostructure Using X-Ray Computed Tomography. *Soil Science*, **171**, 706–718.

Mouginot, P., Prügel, J., Thom, U., Steinhoff, P.O.M., Kupryjanowicz, J. & Uhl, G. (2015) Securing Paternity by Mutilating Female Genitalia in Spiders. *Current Biology*, **25**, 2980–2984.

Mutel, M.H.E., Waugh, D.A., Feldmann, R.M. & Parsons-Hubbard, K.M. (2008) Experimental Taphonomy of Callinectes sapidus and Cuticular Controls on Preservation. *PALAIOS*, **23**, 615–623.

Pagenkemper, S.K., Athmann, M., Uteau, D., Kautz, T., Peth, S. & Horn, R. (2015) The effect of earthworm activity on soil bioporosity - Investigated with X-ray computed tomography and endoscopy. *Soil and Tillage Research*, **146**, 79–88.

Pampush, J.D., Spradley, J.P., Morse, P.E., Harrington, A.R., Allen, K.L., Boyer, D.M. & Kay, R.F. (2016) Wear and its e ff ects on dental topography measures in howling monkeys (Alouatta palliata). *Physical Anthropology*, **161**, 705–721.

Paya, A.M., Silverberg, J.L., Padgett, J. & Bauerle, T.L. (2015) X-ray computed tomography uncovers root – root interactions : Quantifying spatial relationships between ... X-ray computed tomography uncovers root – root interactions : quantifying spatial relationships between interacting root systems in three dimension. *Frontiers in Plant Science*, **29**.

Pelosi, C., Grandeau, G. & Capowiez, Y. (2017) Geoderma Temporal dynamics of earthworm-related macroporosity in tilled and non-tilled cropping systems. *Geoderma*, **289**, 169–177.

Perna, A., Jost, C., Couturier, E., Valverde, S., Douady, S. & Theraulaz, G. (2008) The structure of gallery networks in the nests of termite Cubitermes spp. revealed by X-ray tomography. *Naturwissenschaften*, **95**, 877–884.

Perret, J.S., Al-Belushi, M.E. & Deadman, M. (2007) Non-destructive visualization and quantification of roots using computed tomography. *Soil Biology and Biochemistry*, **39**, 391–399.

Pigneret, M., Mermillod-blondin, F., Volatier, L., Romestaing, C., Maire, E., Adrien, J., Guillard, L., Roussel, D. & Hervant, F. (2016) Urban pollution of sediments : Impact on the physiology and burrowing activity of tubificid worms and consequences on biogeochemical processes. *Science of the Total Environment*, **568**, 196–207.

Porre, R.J., Groenigen, J.W. Van, Deyn, G.B. De, Goede, R.G.M. De & Lubbers, I.M. (2016) Soil Biology & Biochemistry Exploring the relationship between soil mesofauna , soil structure and N 2 O emissions. *Soil Biology and Biochemistry*, **96**, 55–64.

Postnov, A., De Clerck, N., Sasov, A. & Van Dyck, D. (2002) 3D in-vivo X-ray microtomography of living snails. *Journal of Microscopy*, **205**, 201–204.

Rademacher, E., Fahlberg, A., Raddatz, M., Schneider, S. & Voigt, K. (2013) Galenics: Studies of the toxicity and distribution of sugar substitutes on Apis mellifera. *Apidologie*, **44**, 222–233.

Rae, T.C., Vidarsdóttir, U.S., Jeffery, N. & Steegmann, A.T. (2006) Developmental response to cold stress in cranial morphology of Rattus: implications for the interpretation of climatic adaptation in fossil hominins. *Proceedings of the Royal Society B: Biological Sciences*, **273**, 2605–2610.

Renaud, S., Gomes Rodrigues, H., Ledevin, R., Pisanu, B., Chapuis, J.-L. & Hardouin, E.A. (2015) Fast morphological response of house mice to anthropogenic disturbances on a Sub-Antarctic island. *Biological Journal of the Linnean Society*, 513–526.

Rogasik, H., Schrader, S., Onasch, I., Kiesel, J. & Gerke, H.H. (2014) Micro-scale dry bulk density variation around earthworm (Lumbricus terrestris L.) burrows based on X-ray computed tomography. *Geoderma*, **213**, 471–477.

Schönberg, C.H.L. (2001) Estimating the extent of endolithic tissue of a Great Barrier Reef clionid sponge. *Senckenbergiana maritima*, **31**, 29–39.

Schrader, S., Rogasik, H., Onasch, I. & J??gou, D. (2007) Assessment of soil structural differentiation around earthworm burrows by means of X-ray computed tomography and scanning electron microscopy. *Geoderma*, **137**, 378–387.

Schwabe, E., Holtheuer, J. & Schories, D. (2014) First record of a mesoparasite (Crustacea, Copepoda) infesting a polyplacophoran (Mollusca, Polyplacophora) in Chilean waters, with an overview of the family Chitonophilidae (Crustacea & Mollusca). *Spixiana*, **37**, 165–182.

Seidel, R., Lyons, K., Blumer, M., Zaslansky, P., Fratzl, P., Weaver, J.C. & Dean, M.N. (2016) Journal of Anatomy. *Journal of Anatomy*, **229**, 681–702.

Self, C.J. (2015) Dental root size in bats with diets of different hardness. *Journal of Morphology*, **276**, 1065–1074.

Silbiger, N.J., Guadayol, O., Thomas, F.O.M. & Donahue, M.J. (2016) A novel μct analysis reveals different responses of bioerosion and secondary accretion to environmental variability. *PLoS ONE*, **11**, 11–16.

Soné, K., Mori, T., Ide, M., Setoguchi, M. & Yamanouchi, K. (1995) Application of Computer Tomography to Surveys of the Galleries of the Oak Borer, Platypus quercivorus (Maruyama) (Coleoptera: Platypodidae). *Japanese journal of applied entomology and zoology*, **39**, 341–344.

Soons, J., Genbrugge, A., Podos, J., Adriaens, D., Aerts, P., Dirckx, J. & Herrel, A. (2015) Is Beak Morphology in Darwin's Finches Tuned to Loading Demands? *PloS one*, **10**, e0129479.

Tarver, M.R., Shade, R.E., Tarver, R.D., Liang, Y., Krishnamurthi, G., Pittendrigh, B.R. & Murdock, L.L. (2006) Use of micro-CAT scans to understand cowpea seed resistance to Callosobruchus maculatus. *Entomologia Experimentalis et Applicata*, **118**, 33–39.

Tollner, E.W. (1991) X-ray computed tomography applications in soil ecology studies. *Agriculture, Ecosystems and Environment*, **34**, 251–260.

Yunusa, I.A.M., Braun, M. & Lawrie, R. (2009) Amendment of soil with coal fly ash modified the burrowing habits of two earthworm species. *Applied Soil Ecology*, **42**, 63–68.
