# Supplementary material for: X‐ray computed tomography and its potential in ecological research: A review of studies and optimization of specimen preparation
Source: Ecol Evol. 2018 Jul 6;8(15):7717–32. doi: 10.1002/ece3.4149 (PMC6106166; doi:10.1002/ece3.4149)
Supplement: Supplementary file 4 [file ECE3-8-7717-s004.pdf]

**Manufacturer**	**Model/Series**
Perkin Elmer (Caliper LifeSciences)	Quantum GX microCT
	IVIS SpectrumCT
CT imaging	TomoScope® Synergy
FEI	HeliScan microCT
GE Measurement and Control Solutions	phoenix nanotom - microCT & nanoCT
	phoenix v|tome|x - CT
Hitachi Aloka Medical	LaTheta LCT-200 Series
inviscan imaging systems	IRIS PET/CT
Mediso	NanoScan SPECT/CT,
	NanoScan PET/CT
	NanoSPECT/CT
Milabs	U-CT
	U-SPECT/CT
	VECTor/CT
NanoFocus Ray	NFR Polaris-G90
Nikon Metrology	MCT 225
	XT H 225
North Star Imaging	ImagiX
	X25
	X50
Precision X-Ray	X-RAD SmART
RX Solutions	EasyTom
	DeskTom
	UltraTom
Rigaku	nano3DX
	CT Lab
Scanco Medical	µCT 35
	µCT 40
	µCT 50
	µCT 100
	vivaCT 40
	vivaCT 75
	vivaCT 80
	XtremeCT
Sedecal	SuperArgus PET/CT
Bruker microCT	SkyScan 1172
	SkyScan1173
	SkyScan1174
	SkyScan1176
	Skyscan1272
	SkyScan1278
	Skyscan 1294
	SkyScan2011
	Skyscan 2211
Toshiba IT & Control Systems	Toscaner 30000-µhd
TriFoil Imaging	InSyTe CT
	TomoScope® XS

Werth, Inc.	TomoScope® 200
	TomoScope® HV
	TomoCheck® HA
Zeiss	Xradia 520 Versa
	Xradia 510 Versa
	Xradia 410 Versa
	Xradia 810 Ultra
	Xradia 800 Ultra
Xstrahl	SARRP
